# *Nocardia cyriacigeogica* from Bovine Mastitis Induced *In vitro* Apoptosis of Bovine Mammary Epithelial Cells via Activation of Mitochondrial-Caspase Pathway

**DOI:** 10.3389/fcimb.2017.00194

**Published:** 2017-05-18

**Authors:** Wei Chen, Yongxia Liu, Limei Zhang, Xiaolong Gu, Gang Liu, Muhammad Shahid, Jian Gao, Tariq Ali, Bo Han

**Affiliations:** ^1^Department of Veterinary Clinics, College of Veterinary Medicine, China Agricultural UniversityBeijing, China; ^2^Department of Veterinary Clinics, College of Veterinary Medicine, Shandong Agricultural UniversityTai‘an, China

**Keywords:** bovine mastitis, *Nocardia cyriacigeorgica*, bMECs, ultrastructural feature, mitochondria-dependent apoptotic pathway

## Abstract

*Nocardia* is one of the causing agents of bovine mastitis and increasing prevalence of nocardial mastitis in shape of serious outbreaks has been reported from many countries. However, the mechanisms by which this pathogen damages the bovine mammary epithelial cells (bMECs) is not yet studied. Therefore, this study was designed with the aim to evaluate the apoptotic effects elicited by *Nocardia* and to investigate the pathway by which the *Nocardia* induce apoptosis in bMECs. Clinical *Nocardia cyriacigeorgica* strain from bovine mastitis was used to infect the bMECs for different time intervals, *viz*. 1, 3, 6, 12, and 18 h, and then the induced effects on bMECs were studied using adhesion and invasion assays, release of lactate dehydrogenase (LDH), apoptosis analysis by annexin V and propidium iodide (PI) double staining, morphological, and ultrastructural observations under scanning electron microscope (SEM) and transmission electron microscope (TEM), mitochondrial transmembrane potential (ΔΨm) assay using flow cytometry, and the protein quantification of mitochondrial cytochrome c and caspase-9 and caspase-3 by western blotting. The results of this study showed that *N. cyriacigeorgica* possessed the abilities of adhesion and invasion to bMECs. *N. cyriacigeorgica* was found to collapse mitochondrial transmembrane potential, significantly (*p* < 0.05) release mitochondrial cytochrome c and ultimately induce cell apoptosis. Additionally, it promoted casepase-9 (*p* < 0.01) and casepase-3 (*p* < 0.05) levels, significantly (*p* < 0.01) increased the release of LDH and promoted DNA fragmentation which further confirmed the apoptosis. Furthermore, *N. cyriacigeorgica* induced apoptosis/necrosis manifested specific ultrastructure features under TEM, such as swollen endoplasmic reticulum, cristae degeneration, and swelling of mitochondria, vesicle formation on the cell surface, rupturing of cell membrane and nuclear membrane, clumping, fragmentation, and margination of chromatin. The present study is the first comprehensive insight into patho-morphological ultrastructural features of apoptosis/necrosis induced by *N. cyriacigeorgica*, which concluded that the clinical *N. cyriacigeorgica* induced apoptotic changes in the bMECs through mitochondrial-caspase dependent apoptotic pathway.

## Introduction

*Nocardia* species are gram-positive, aerobic, saprophytic, and widespread environmental actinomycetes, which have been reported as an opportunistic intracellular pathogen of human and animals (Sullivan and Chapman, 2010; Conville and Witebsky, 2011). *Nocardia* can cause localized or systemic nocardiosis with purulency or granulomas (Holland, 2010), which is probably transmitted by inhalation, ingestion or traumatic implantation, and can be disseminated through lymph and blood circulation (Ambrosioni et al., 2010). The most important species causing nocardiosis include *N. cyriacigeorgica, N. asteroides, N. brasiliensis, N. farcinica, and N. nova* (Ribeiro et al., 2008; Liu et al., 2011; Condas et al., 2013; Brown-Elliott et al., 2015; Hashemi-Shahraki et al., 2015). In human beings, the common manifestations of nocardiosis are pulmonary nocardiosis, central nervous system (CNS) nocardiosis, extrapulmonary nocardiosis, cutaneous, subcutaneous or lymphocutaneous nocardiosis, and nocardial bacteremia (Ambrosioni et al., 2010; Al Akhrass et al., 2011; Wilson, 2012). Whereas, in cattle, it is associated with farcy, abortion, pulmonary, and systemic nocardiosis (Beaman and Sugar, 1983; Bawa et al., 2010; Hamid, 2012). Nocardial bovine mastitis is the most important manifestation of nocardiosis and it has been reported from many countries (Dohoo, 1989; Hamid et al., 1998; Cook and Holliman, 2004; Brown et al., 2007; Pisoni et al., 2008; Ribeiro et al., 2008; Condas et al., 2013). Nocardial mastitis is characterized by the suppurative or granulomatous inflammation of the mammary gland followed an acute or chronic course (Bättig et al., 1989; Pisoni et al., 2008; Ribeiro et al., 2008). Moreover, its huge economic losses are mostly due to decrease milk production and culling of dairy cows (Cook and Holliman, 2004; Condas et al., 2013).

Bacterial adhesion and invasion are considered as important pathogenetic and virulence factors in the infection processes (Dego et al., 2002). Several *in vivo* and *in vitro* experiments demonstrated that *Nocardia* possessed the abilities to adhere to and invade into various types of cells, inducing cellular and tissue damages (Beaman and Beaman, 1998; Chapman et al., 2003; Beaman and Tam, 2008; Kohbata et al., 2009). When *Nocardia* attached to and rapidly penetrated through capillary endothelial cells (Beaman and Ogata, 1993), then entered the brain parenchyma, eliciting Lewy body inclusion in brain and Parkinson's symptoms in experimental animals (Chapman et al., 2003; Beaman and Tam, 2008). A previous study reported that *Nocardia* infection may induce macrophages and dendritic cells to differentiate into foamy cells (Meester et al., 2014). Furthermore, the invasion of *Nocardia* can even lead to the prevention of phagosome-lysosome fusion), inhibition of proteasome activity (Barry and Beaman, 2007), resistance to oxidative killing, blockage of phagosomal acidification, and alteration of lysosomal enzyme activity in macrophages (Beaman and Beaman, 1994). *Staphylococcus aureus* adhesion and invasion to bovine mammary epithelial cells (bMECs) has been proven to be the key events in the pathogenesis of bovine mastitis and the infected cells exhibited apoptotic morphology (Bayles et al., 1998; Dego et al., 2002); but for *Nocardia*, the adhesion and invasion ability to bMECs and the cell death effects are still unclear.

Cell death, the ultimate consequence of injury in host cells infection mainly includes apoptosis and necrosis. Necrosis is characterized by loss of cell membrane integrity, release of cellular contents and motivating inflammatory reaction; whereas, apoptosis is generally developed with cell membrane integrity, internucleosomal DNA fragmentation, formation of apoptotic bodies, no inflammatory reaction, and mediated through the intrinsic and extrinsic apoptosis pathway (Lamkanfi and Dixit, 2010). The extrinsic apoptosis pathways involve death receptors and caspase-8 signaling; whereas the intrinsic apoptotic pathway mainly targets the mitochondria (Lamkanfi and Dixit, 2010; Galluzzi et al., 2012).

In many bacterial pathogens, mitochondria-dependent apoptotic pathways have been well-recognized as a major pathogenic strategy (Ashida et al., 2011). During the process, apoptotic factors opens the mitochondrial permeability transition pore that ultimately result in loss of membrane potential and activation of cytochrome c (Yang et al., 2015; Xu et al., 2016). Then cytochrome c binds to apoptotic protease-activating factor I (Apaf-I) and tempts oligomerization. Consequently, apoptosome complex form by assembling of Apaf-1 oligomers with procaspase-9. Caspase-9 subsequently triggers apoptosis by caspase-3 activation (Elmore, 2007; Lamkanfi and Dixit, 2010). However, the specific mechanism through which capsase-9 activation triggers apoptosis in response to *N. cyriacigeorgica* in bMECs is unclear. *Nocardia* was shown to induce apoptotic death in dopaminergic cells, PC12 cells and HeLa cells; meanwhile, disruption of the mitochondrial membrane potential and caspase activation were involved in the apoptosis of HeLa cells (Barry and Beaman, 2007). However, the cell death effect of *Nocardia* on bMECs and the specific mechanisms involved in response to nocardial infection remain unknown.

Although, most of studies on *Nocardia* infections in various cells and laboratory animals were performed to demonstrate the pathogenicity and pathogenic mechanisms in central nervous system, respiratory system, and skin or cutaneous tissues (Barry and Beaman, 2007; Beaman and Tam, 2008; Meester et al., 2014; Lira et al., 2016). Nevertheless, there are rare studies focused on pathogenicity and mechanism underlying bovine mastitis caused by *N. cyriacigeorgica*. Therefore, the current study was designed with hypothesis that the *Nocardia* could adhere to and invade into bMECs, inducing apoptotic and necrotic cell death; in addition, *Nocardia* may regulate the cell apoptosis via mitochondrial-caspase pathway.

## Materials and methods

### Cell culture

The bMECs line MAC-T was used in this study which was purchased from Shanghai Jingma Biological Technology Co., Ltd. China. Cells were cultured in DMEM/F-12 (HyClone, USA) supplemented with 10% heat-inactivated Gibco® Fetal Bovine Serum (FBS; HyClone, USA), 100 U/mL penicillin and 100 μg/mL streptomycin at 37°C with 5% CO_2_. Cells for adhesion and invasion assay were cultured in DMEM/F12 medium without antibiotics and while for other assays bMECs were cultured in DMEM/F12 medium with 4% FBS without antibiotics.

### Bacterial culture

*N. cyriacigeorgica* isolated previously from bovine mastitis was activated from frozen stocks by culture on tryptose soya agar (Difco™, Becton Dickison, Sparks, MD USA) supplemented with 5% defibrinated sheep blood and incubated at 37°C for 72 h, then sub-cultured in 7H9 broth to mid-log phase for the following experiments.

### Adhesion assay

Adhesion assay of clinical *N. cyriacigeorgica* to bMECs was performed according to previously described protocols (Pöhlmann-Dietze et al., 2000; Pereyra et al., 2016), with slight modifications as bMECs were infected with *N. cyriacigeorgica* at a multiplicity of infection (MOI, ratio of *N. cyriacigeorgica* to cells) of 50:1 for 10 min, 1, 2, and 3 h at 37°C with 5% CO_2_. After incubation, the supernatants of infected cells were removed and cells were washed three times with phosphate buffer saline (PBS, pH 7.4) to remove non-adherent bacteria. Subsequently, cells were lysed by 1 mL PBS and 1 mL 1% Triton X-100 (0.5% v/v) to release adherent *Nocardia*. In control groups, both *Nocardia* suspensions (1 mL) and the cells were also treated with 1 mL Triton X-100. Finally, cell lysates and treated *Nocardia* supernatants were 10-folds serially diluted, plated onto sheep blood agar plates and incubated at 37°C for 48 h for enumeration of colony forming units (CFU). Adhesion rate of total *Nocardia* was expressed as:

$$\begin{array}{l} \text{Adhesion rate of total }Nocardia\;=\;\frac{\text{Lysate of infected cells (CFU/mL)}}{\text{Lysate of }Nocardia\text{ supernatant and infected cells (CFU/mL)}}\;\times \;100 \end{array}$$

The adhesion assay was repeated four times and each experiment was performed in quadruplicate.

### Invasion assay

In our previous study, we tested several dilutions of amikacin (25, 50, 75, and 100 μg/mL) with high concentration of *Nocardia* (1 × 10^7^ CFU/mL) and with different incubation time (1, 2, and 3 h). As a result, we found that 50 μg/mL of amikacin was enough to kill all *Nocardia* within 2 h; thus, this concentration of amikacin and incubation time was used for the following invasion assay. Invasion assay of *N. cyriacigeorgica* was performed according to the previous method with minor modifications (Beaman and Beaman, 1998; Pereyra et al., 2016). The bMECs were infected with *N. cyriacigeorgica* at a MOI of 50:1 for 10 min, 1, 2, and 3 h. Following incubation, cells were washed three times with PBS and treated with amikacin (50 μg/mL) for 2 h to kill extracellular *Nocardia*. The infected cells without amikacin treatment were used as control group. Then, cells were washed three times with PBS to remove non-adherent bacteria, further lysed with 0.5% Triton X-100 (v/v). Finally, cell lysates were 10-folds serially diluted for CFU determination. Invasion rate of adhered *Nocardia* was expressed as:

$$\text{Invasion rate of adhered }Nocardia\;=\;\frac{\text{Lysate of infected cells with amikacin treatment (CFU/mL)}}{\text{Lysate of infected cells without amikacin treatment (CFU/mL)}}\times 100$$

Invasion rate of total *Nocardia* was expressed as:

$$\begin{array}{l} \text{Invasion rate of total Nocardia}=\\ \text{ Adhesion rate of total }Nocardia\;\times \\ \text{ Invasion rate of adhered }Nocardia \end{array}$$

The invasion assay was repeated four times and each experiment was performed in quadruplicate.

### Lactate dehydrogenase (LDH) release assay

LDH, an enzyme, normally present in the cytoplasm and could release into the cell culture medium through damaged cell membrane after bacterial infection (Loeffler et al., 2004; Viguier et al., 2009). LDH release assay can be used to evaluate cytopathic effect of *N. cyriacigeogica* on bMECs. Cells were infected with *N. cyriacigeorgica* at a MOI of 5:1 for 1, 3, 6, 12, and 18 h at 37°C with 5% CO_2_. In addition, *Nocardia* suspensions in DMEM/F12 with 4% FBS and non-infected cells incubated for the same time were used as control. After incubation, the supernatants were collected and centrifuged at 18,000 g for 15 min. The supernatants were collected again for measurement of LDH release by cytotoxicity LDH Assay Kit-WST® (Dojingdo Laboratories, Kumamoto, Japan). The absorbance at 490 nm was measured by a microplate reader.

### DNA ladder analysis

As a characteristic feature for apoptotic cell death, DNA ladder was applied for the verification of cell apoptosis. The bMECs were infected with *N. cyriacigeorgica* in the same manner as described above. Moreover, cells without *Nocardia* treatment were used as control. Fragmented DNA was collected from cells according to the manufacturer's instructions of the DNA Ladder Extraction Kit (Beyotime, China). Moreover, this kit was also used to extract the DNA of *N. cyriacigeorgica* to determine the interference effect of *N. cyriacigeorgica* DNA. Finally, the DNA samples were electrophoresed on a 1% agarose gel, stained with ethidium bromide, and photographed by gel documentation system (Alpha® Imager EC, SAN LEADRO, USA).

### Apoptosis/necrosis analysis by annexin V/propidium iodide (PI) double staining

Cell death was detected by the FITC Annexin V Apoptosis Detection Kit I (BD, USA) according to the manufacturer's instructions. *N. cyriacigeorgica* were incubated with bMECs at a MOI of 5:1 for 1, 3, 6, 12, and 18 h at 37°C with 5% CO_2_. Cells without *Nocardia* treatment were used as the control groups. After incubation, cells were harvested, washed twice with cold PBS and re-suspended in binding buffer at a concentration of 1 × 10^6^ cells/mL, then 100 μL of the cell suspension was stained by 5 μL FITC Annexin V and 5 μL PI. Following incubation for 15 min at room temperature, the samples were analyzed by the BD FacsCalibur flow cytometer (New Jersey, USA) within 1 h.

### Scanning electron microscopy (SEM) and transmission electron microscopy (TEM)

The bMECs adhered on coverslips were washed three times with cold PBS and fixed with 2.5% glutaraldehyde at 4°C for 1.5 h After washing, cells were dehydrated through a graded series of ethanol (30, 50, 70, 80, 90, 100, and 100% ethanol) for 15 min in each at room temperature. Subsequently, cells were immersed into tert-butyl alcohol for 30 min. After lyophilization and gold coating, these cell samples were observed on a scanning electron microscope (Hitachi S-3000N, Japan).

For TEM, cells were harvested and the pretreatment of the cells before dehydration was similar to that for SEM. After dehydration by graded ethanol and acetone (three changes, for 10 min each), cells were sequentially embedded in epoxy resin-acetone mixtures (2:1) for 2 h and in pure resin overnight at 37°C. When the resin had polymerized, ultra-thin sections were cut by an ultramicrotome (Leica EM, Germany), stained with 1% uranyl acetate followed by lead citrate and viewed on a transmission electron microscope (Hitachi H-7650, Japan).

### Mitochondrial transmembrane potential (ΔΨM) assay

The bMECs were infected with *N. cyriacigeorgica* at a MOI of 5:1 for 1, 3, 6, 12, and 18 h at 37°C with 5% CO_2_. Cells were collected for mitochondrial damage detection according the changes of ΔΨm. The ΔΨm was measured using a mitochondrial membrane potential assay kit with JC-1 (Beyotime, China) by flow cytometry. JC-1 was a dual-emission potential-sensitive probe and formed red-fluorescent aggregates in the mitochondria of the cells with higher potentials. But membrane potential collapse could result in the failure to red JC-1 in the mitochondria and the dye return to green-fluorescent monomer.

### Western blot analysis

The bMECs were infected with *N. cyriacigeorgica* at a MOI of 5:1 for 1, 3, 6, 12, and 18 h at 37°C with 5% CO_2_. Total proteins and cytoplasmic proteins without mitochondrion were respectively extracted from nocardial infected cells with RIPA lysis buffer (Beyotime, China) and Cell Mitochondria Isolation Kit (Beyotime, China). The protein concentrations were detected by the BCA method. Equivalent proteins from each sample were separated by SDS-PAGE gel, and then transferred onto PVDF membranes. Subsequently, the membranes were blocked in 5% BSA and incubated with the primary antibody for Cytochrome C (1:200, Santa, USA), caspase-9 (1:500, Santa, USA), caspase-3 (1:100, Santa, USA), and tubulin (1:1,000, Cell Signaling Technology, USA) overnight at 4°C followed by incubation with HRP-conjugated secondary antibody (1:5,000) for 1 h at room temperature. Finally, the bands were visualized using a BeyoECL Plus ECL Kit (Beyotime, China). Densitometric analysis of the bands was quantified using Image J and these results were normalized using β-actin.

### Apoptosis analysis in parallel with the growth of intracellular *N. cyriacigeorgica*

This experiment was carried out to evaluate the apoptosis and intracellular growth of *Nocardia* by killing the extracellular bacteria. The bMECs were infected with *N. cyriacigeorgica* at a MOI of 5:1 in DMEM/F12 for 2 h. Following co-cultures, cells were washed with PBS and treated with amikacin (50 μg/mL in DMEM/F12) for 2 h to kill extracellular *Nocardia*. Then, cells were washed three times with PBS and cultured in DMEM/F12 medium with 4% FBS for 0 h (control group), 1, 3, 6, 12, and 18 h at 37°C with 5% CO_2_ for the following steps: (i) for cell apoptotic analysis, cells were collected, after incubation, for apoptotic analysis by the FITC Annexin V Apoptosis Detection Kit I (BD, USA) as previously described; (ii) for total bacterial counting, the medium (1 mL) with infected bMECs was treated with 1 mL 1% Triton X-100 (v/v) for the CFU enumeration of total *Nocardia*. Simultaneously, the parallel infected bMECs were washed three times with PBS, further lysed with Triton X-100 and 10-folds serially diluted for CFU determination of the intracellular *Nocardia*. The number of *Nocardia* was expressed as Log_10_ CFU/Well. The bacterial counting was repeated four times and each experiment was performed in quadruplicate; (iii) for observation of bacteria Gram staining was carried out. After washing with PBS, cells adhered on coverslips were fixed with 4% paraformaldehyde for 20 min and then permeabilized with 0.2% Triton X-100 (v/v) for 10 min at room temperature. Finally, cells were washed, stained by Gram staining and observed under light microscope (Olympus, Japan).

### Data interpretation

Each experiment was repeated at least three times and the data were expressed as mean ± standard deviation (*SD*). Statistical differences between groups were analyzed by One-way ANOVA followed by the Duncan and LSD multiple tests using SPSS 20.0 (SPSS, Inc., Chicago, IL, USA). *P*-value < 0.05 was regarded as statistically significant.

## Results

### Adhesion and invasion assay

The preliminary quantitative study is shown in Table 1. The adhesion rate of total *N. cyriacigeorgica* increased from 4.5 to 42.8% within 3 h and this increase was time-dependent. Whereas, the invasion rate of total *Nocardia* also showed a time-dependent increase from 3.3 to 33.35% within 3 h. On the other hand, the invasion rate of adhered bacteria was persistent at a range of 64.4 to 77.9% within 3 h, indicating that more than 60% of the adhered *Nocardia* can invade into cells. Remarkably, these data showed the rapid adhesive and invasive capabilities of *N. cyriacigeorgica* with bMECs (Table 1).

**Table 1:** **Adhesion and invasion rate of *N. cyriacigeorgica* to bMECs**.

**Item**	**Rate (%)**			
	**10 min**	**1 h**	**2 h**	**3 h**
Adhesion rate of total *Nocardia*	4.5 ± 0.7	22.0 ± 2.6	39.8 ± 2.4	42.8 ± 1.8
Invasion rate of adhered *Nocardia*	73.6 ± 8.0	64.4 ± 8.4	70.5 ± 8.5	77.9 ± 7.9
Invasion rate of total *Nocardia*	3.4 ± 0.8	14.3 ± 3.0	28.2 ± 4.9	33.4 ± 4.1

*Invasion rate of total Nocardia = Adhesion rate of total Nocardia × Invasion rate of adhered Nocardia*.

### LDH release assay

**Figure 1:**
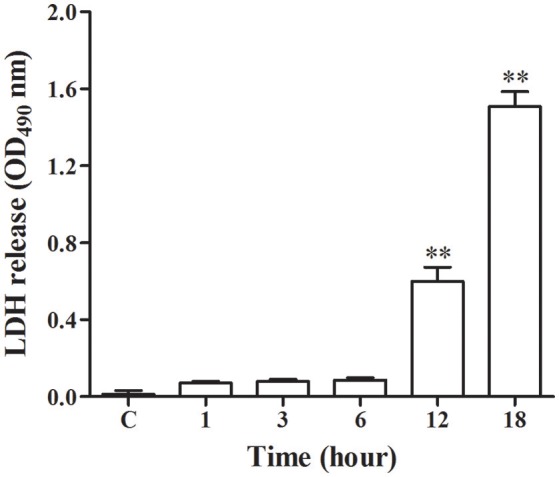
**Lactate dehydrogenase (LDH) release assay in the medium**. The LDH release assay was repeated three times and each experiment was performed in triplicate. Results were presented as Mean ± SD. ^**^P < 0.01 as compared to the control group.

As shown in Figure 1, there was release of LDH at 1, 3, and 6 h post-infection, however at 12 and 18 h post-infection there was a significant increase (*p* < 0.01) of LDH as compared with control group. This indicated that *N. cyriacigeorgica* seriously damage the cell membranes of bMECs with the passage of time.

### DNA ladder assessment

**Figure 2:**
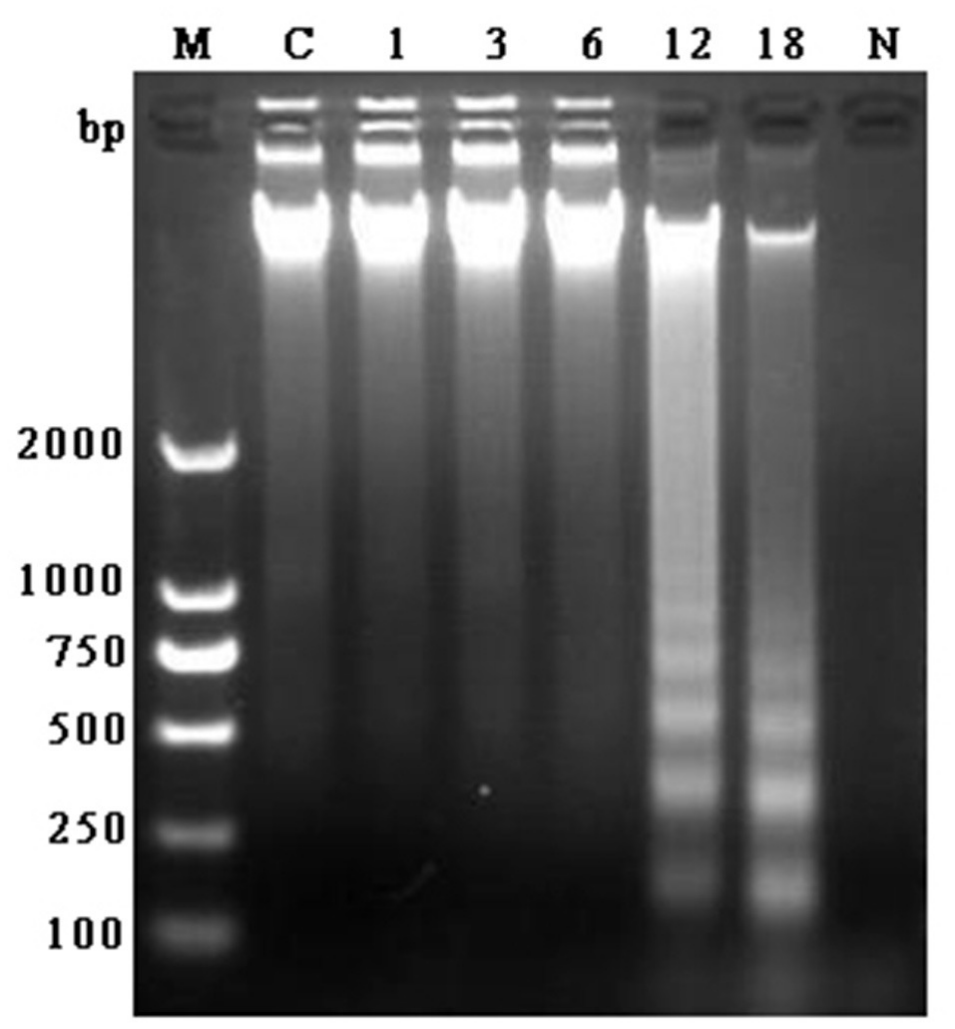
**Agarose gel of electrophoresis of DNA fragment obtained from bMECs at 1, 3, 6, 12, and 18 h post-infection, control group and *N. cyriacigeorgica***. M, Marker; C, Control group; 1–18, Cells infected with *N. cyriacigeorgica* for 1, 3, 6, 12, and 18 h; N, *N. cyriacigeorgica*.

DNA fragment is shown in Figure 2. There was no appearance of DNA ladder band in the bMECs of control group, 1, 3, and 6 h groups and also in *N. cyriacigeorgica* control group. But in the infected groups at 12 and 18 h, distinct typical DNA ladder bands were observed. These results indicated that *N. cyriacigeorgica* could induce apoptosis/necrosis in bMECs and the apoptotic DNA fragment was observed after 12 h post-infection.

### Apoptosis/necrosis analysis

**Figure 3:**
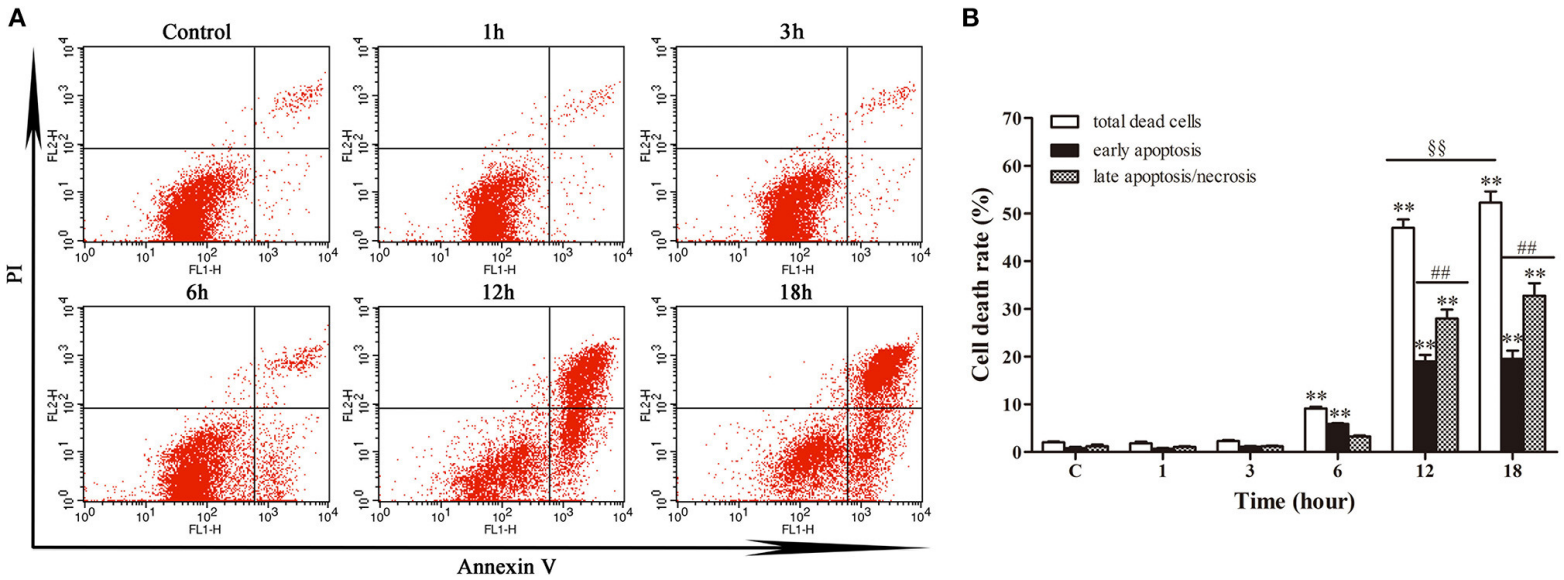
**Apoptosis and necrosis of bMECs analyzed by flow cytometry with annexin V/propidium iodide (PI) double staining. (A)** Two dimensional scatter plots of FITC Annexin V vs. PI through flow cytometry. Cells stained negative for FITC Annexin V and PI in the lower left quadrant shows alive cells. Cells stained positive for FITC Annexin V and negative for PI in the lower right quadrant are representing early apoptosis. Cells stained positive for both FITC Annexin V and PI in the upper right quadrant are the late apoptotic/necrotic cells. **(B)** Percentage of early apoptotic cells and late apoptotic/necrotic cells. Data were presented as Mean ± *SD* of three independent experiments. ^**^P < 0.01 as compared with the control group. ^*##*^*P* < 0.01 as compared between the rate of early and late apoptosis/necrosis at the same time point. ^§§^*P* < 0.01 indicates the significant differences in total cells death rate between 12 and 18 h.

Annexin V/PI double staining was used to analyze the early apoptotic cells and late apoptotic/necrotic cells. Figure 3 depicted limited apoptosis/necrosis at 1 and 3 h post-infection. The early apoptosis rate was increased significantly (*p* < 0.01) from 6 h post-infection compared with control group. There was a slight increase of apoptosis at 6 h post-infection; then the apoptotic/necrotic changes increased dramatically (*p* < 0.01) at 12 and 18 h post-infection in comparison with the control group. Furthermore, both at 12 h and 18 h, the late apoptosis/necrosis rate was much higher (*p* < 0.01) than early apoptosis rate. Additionally, the total death rate at 18 h (52.26%) was significantly higher (*p* < 0.01) than that at 12 h (46.99%). These data suggested that *N. cyriacigeorgica* could cause both apoptosis and necrosis, which was seriously intensified in time dependent manner.

### Scanning electron microscopy (SEM)

**Figure 4:**
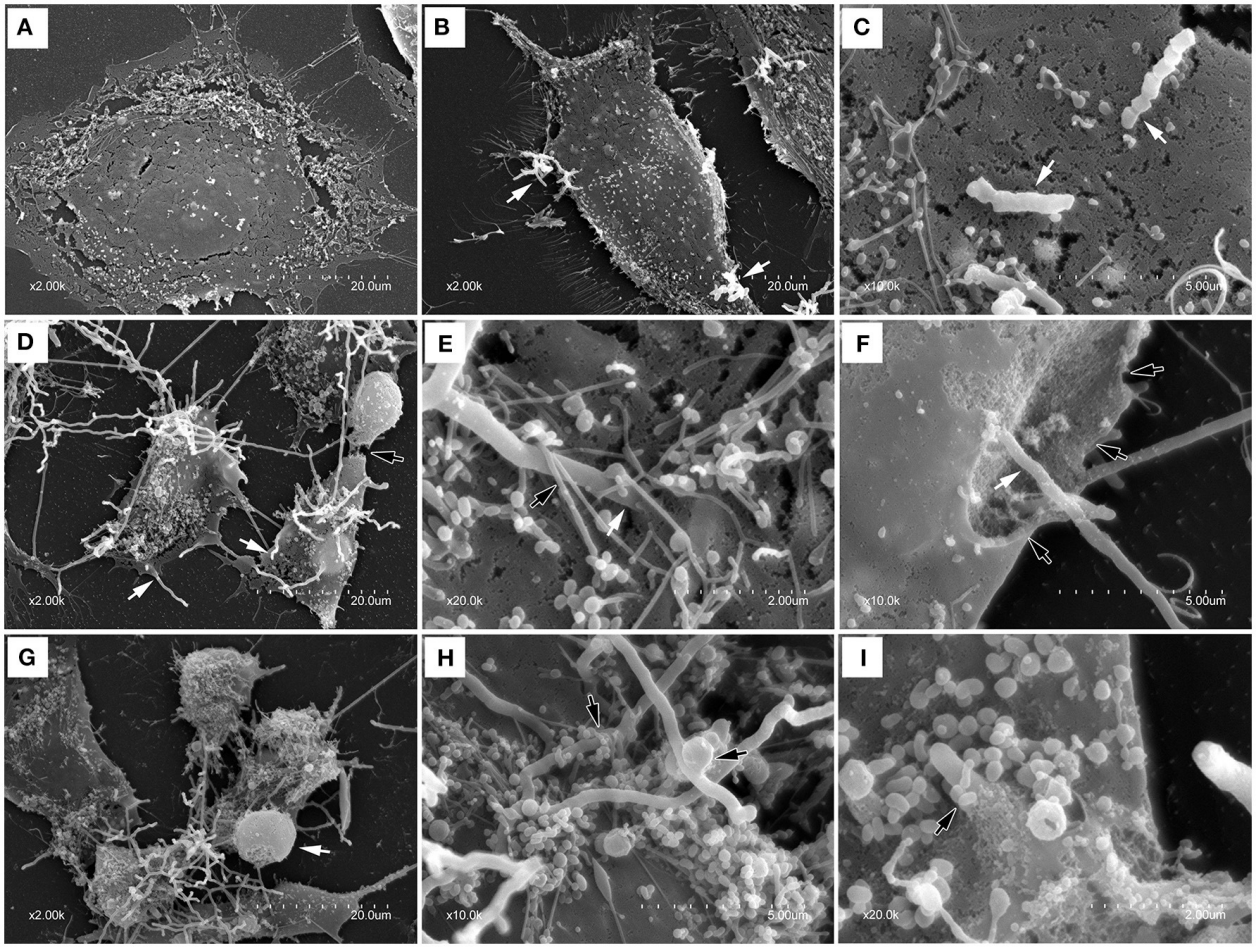
**Scanning electron photomicrographs showing interaction of bMECs infected with *N. cyriacigeorgica*. (A)** demonstrates the normal cell without *N. cyriacigeorgica* infection that adherent to coverslip. **(B)** The cell with folding edge and several *N. cyriacigeorgica* filaments (white arrows) adhered on the cell surface at 6 h. **(C)** Apical attachment of *N. cyriacigeorgica* to cell surface at 6 h. **(D)** Atrophic and fragmented cells (black arrow) following long *N. cyriacigeorgica* filaments throughout cells (white arrows) at 12 h. **(E)** The white arrow showing *N. cyriacigeorgica* filament penetrating into the cell membrane and the black arrow presenting microvilli trapping *N. cyriacigeorgica* filament at 12 h. **(F)** A gap (black arrows) on the cell membrane with *N. cyriacigeorgica* filaments inside it (white arrows) at 12 h. **(G)** Shrinking cells coated with numerous *N. cyriacigeorgica* filaments. A nucleus-like spherical protrusion observed on the cell surface (white arrow) at 18 h. **(H)** Microvilli wrapped *N. cyriacigeorgica* filaments at 18 h. **(I)** A tip of intracellular *N. cyriacigeorgica* filament penetrated through the cell membrane at 18 h.

To further confirm the adhesion, invasion, LDH release and cell death, SEM was used to test the interaction of bMECs infected with *N. cyriacigeorgica* and the cell damages. There were no morphological changes in control group (Figure 4A). At 6 h post-infection, morphological changes were observed as mild disruption of the some bMECs and short *Nocardia* filaments adhered on the surface of cells (Figures 4B,C). At 12 and 18 h post-infection, bMECs were obvious shrinkage, cytomorphosis, desquamation, and cell membrane breakage, with spherical protrusion appearance on some cell surface, abundant growing mycelium covering the cells and filaments penetrating into cell membrane both from the extracellular and intracellular (Figures 4D–I). Particularly, at 6, 12, or 18 h, SEM results demonstrated that microvilli on the cell surface were totally wrapped by *Nocardia* filaments (Figures 4E,H,I). Thus, these changes to the cellular morphology suggested that the penetration and viability of *N. cyriacigeorgica* may be responsible for aforesaid cell damages.

### Transmission electron microscopy (TEM)

**Figure 5:**
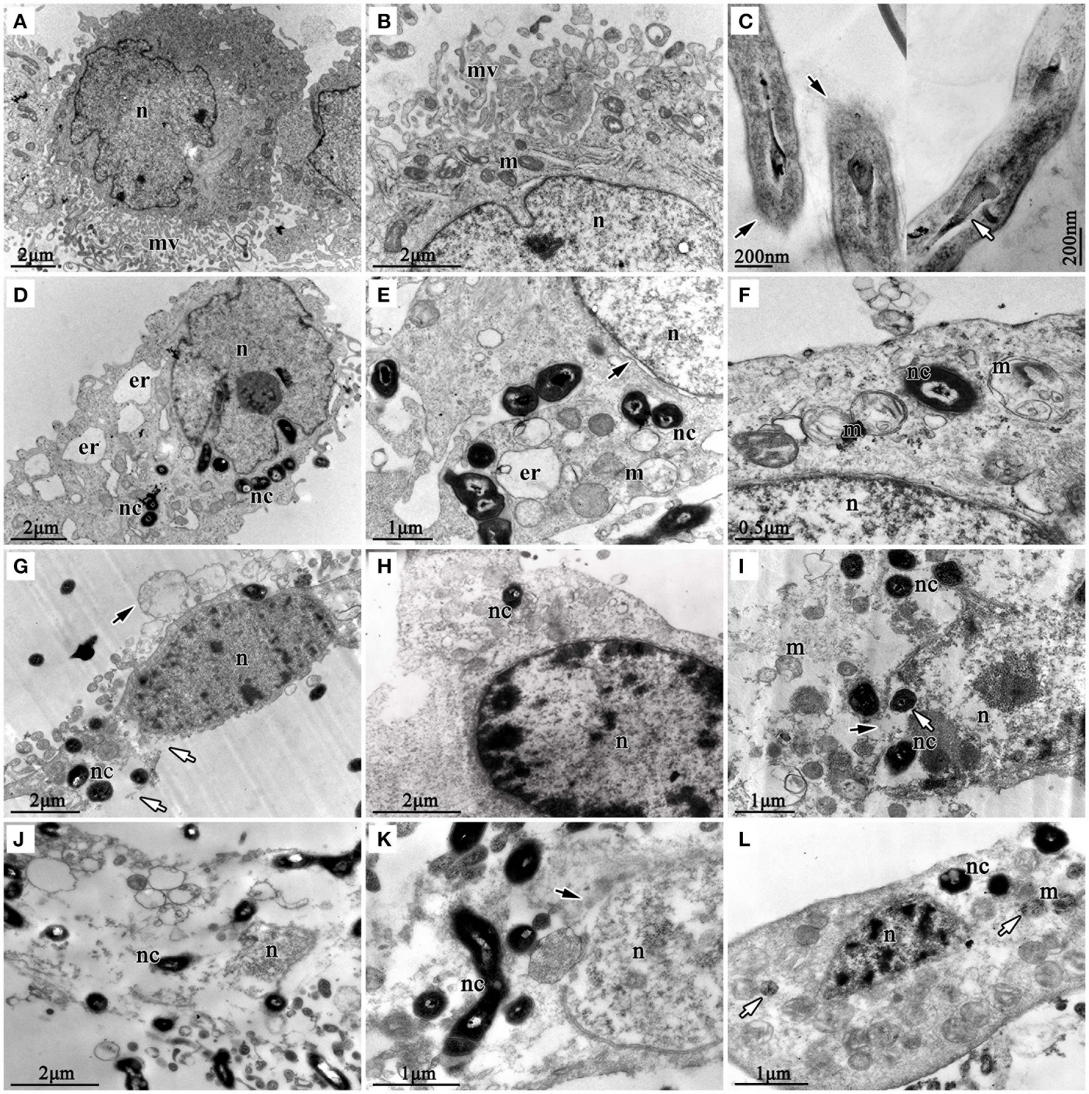
**Transmission electron photomicrographs showing ultrastructural pathological changes in bMECs co-cultured with *N. cyriacigeorgica*. (A,B)** are the non-infected cells that show rich microvilli on cell surface and abundant mitochondria in cytoplasm, non-infected cells. **(C)** is the longitudinal sections of *N. cyriacigeorgica* incubated in DMEM/F12 with 4% FBS which depict the tips of log-phase *N. cyriacigeorgica* presenting hazy cell wall (black arrows) compared with that in the trunk. The white arrow is showing tubular-like nucleus of *N. cyriacigeorgica*. **(D)** Nocardial cells in cytoplasm and swollen endoplasmic reticulum at 6 h. **(E)** Flocculent densities in swollen and round mitochondria, expansion of perinuclear space (black arrow) at 6 h. **(F)** Mitochondria with electron density decreasing and crista degeneration at 6 h. **(G)** Vesicles on the cell surface (black arrow), chromatin condensation and fragmentation, cell membrane breakage (white arrows) at 12 h. **(H)** Chromatin condensation and accumulation at 12 h. **(I)** Mitochondria disruption, nuclear membrane rupture (black arrow); nocardial cells in cell nucleus (white arrow) at 12 h. **(J)** Cell disruption with loss of organelles at 18 h. **(K)** The nuclear membrane ruptures and disruption (black arrow) at 18 h. **(L)** Mitochondrial pyknosis and intramitochondrial dense granules accumulation, chromatin condensation and fragmentation, loss of microvilli (white arrow) at 18 h. *nc*-*N. cyriacigeorgica, n*-nucleus, *m*-mitochondrion, *mv*-microvillus, *er*-endoplasmic reticulum.

TEM was used to check the internalization of nocardial cells and ultrastructural changes in bMECs with *N. cyriacigeorgica* infection. The ultrastructure changes of infected cells are elaborated in Figure 5, which included swollen endoplasmic reticulum, cristae degeneration and swelling of mitochondria and also expansion of perinuclear space at 6 h post-infection (Figures 5D–F); while at 12 h of post infection, vesicle formation on the cell surface, rupturing of cell membrane, and nuclear membrane, chromatin clumping, fragmentation and margination of chromatin were noted (Figures 5G–I). Finally, serious disruption of cells, loss of organelles, and intramitochondrial dense granules accumulation were observed, at 18 h post-infection (Figures 5J–L). Furthermore, *Nocardial* cells were found both in cytoplasm and nucleus. These observations showed a process of progressive cell necrosis induced by clinical *N. cyriacigeorgica* infection.

### Mitochondrial transmembrane potential (ΔΨm) assay

**Figure 6:**
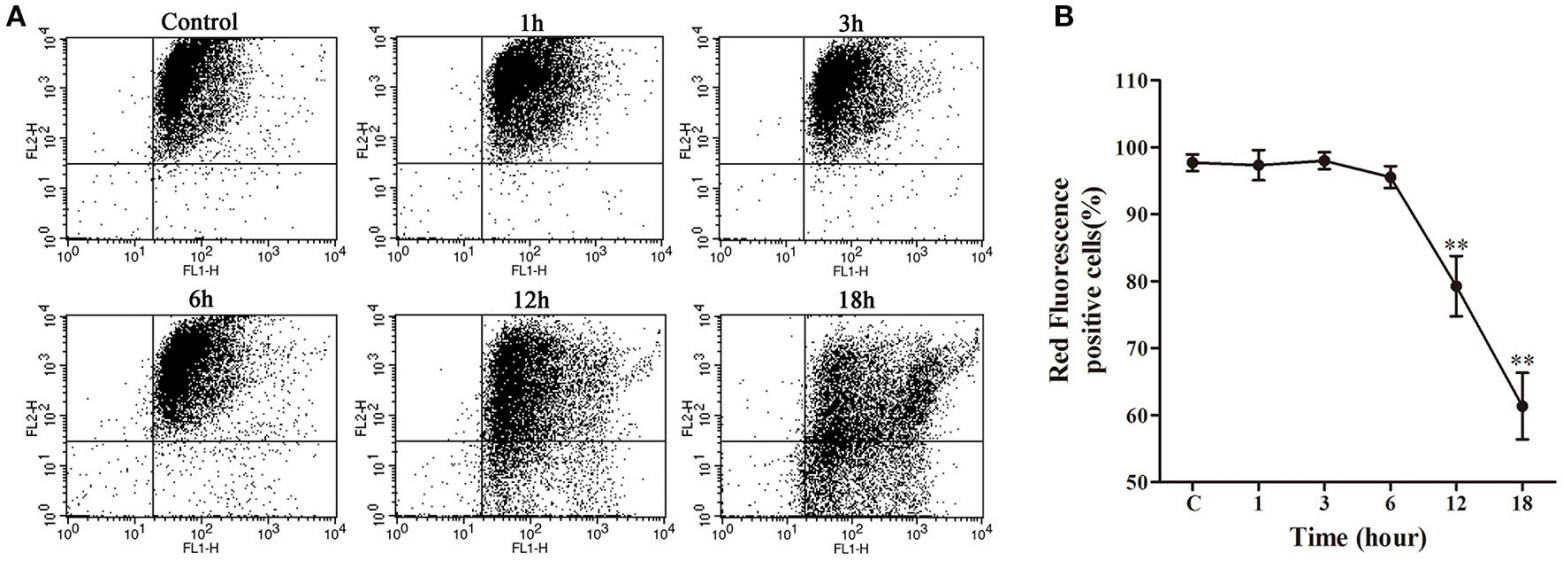
**Mitochondrial transmembrane potential (ΔΨm) assay of bMECs co-cultured with *N. cyriacigeorgica*. (A)** ΔΨm determined using JC-1 via flow cytometry. Red fluorescence positive cells in the upper right quadrant and green fluorescence positive cells in the lower right quadrant. **(B)** The percentage of red fluorescence positive cells. Results were presented as Mean ± *SD* of three independent experiments. ^**^P < 0.01 as compared to the control group.

Following a relatively stable state of ΔΨm in the early stage (1–6 h) of nocardial infection, there was a remarkable decrease (*p* < 0.01) of ΔΨm in the late stage of infection *viz*. 12–18 h in comparison with the control group (as shown in Figure 6).

### Western blot analysis

**Figure 7:**
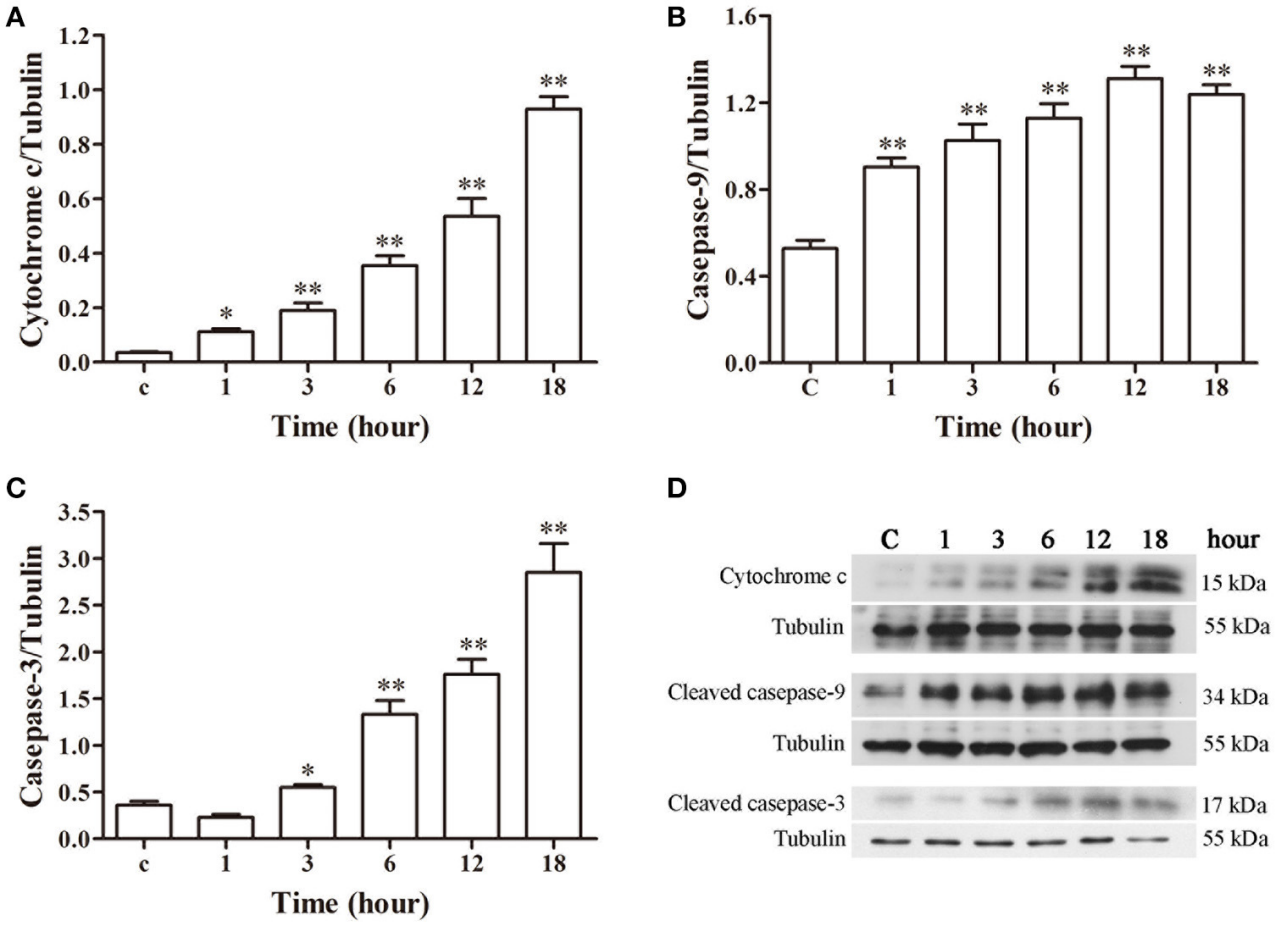
**Protein expression assay of bMECs incubated with *N. cyriacigeorgica* by western blot analysis. (A)** The release of mitochondrial cytochrome c. **(B)** The expression of cleaved caspase-9. **(C)** The expression of cleaved caspase-3. **(D)** The western blot assays of cytochrome c, cleaved caspase-9 and cleaved caspase-3 expression at 1, 3, 6, 12, and 18 h after *N. cyriacigeorgica* infection in bMECs. Results were presented as Mean ± *SD* of three independent experiments. ^*^P < 0.05, ^**^P < 0.01 as compared with the control group.

The western blotting results of cytochrome c, caspase-9 and caspase-3 are depicted in Figure 7. A gradual increase in mitochondrial cytochrome c release was observed after 1 h post-infection (*p* < 0.05), with significantly higher caspase-9 activation (*p* < 0.01) from 1 h and markedly enhanced caspase-3 (*p* < 0.05) activation from 3 h of infection as compared to the control group.

### Apoptosis analysis in parallel with the growth of intracellular *N. cyriacigeorgica*

**Figure 8:**
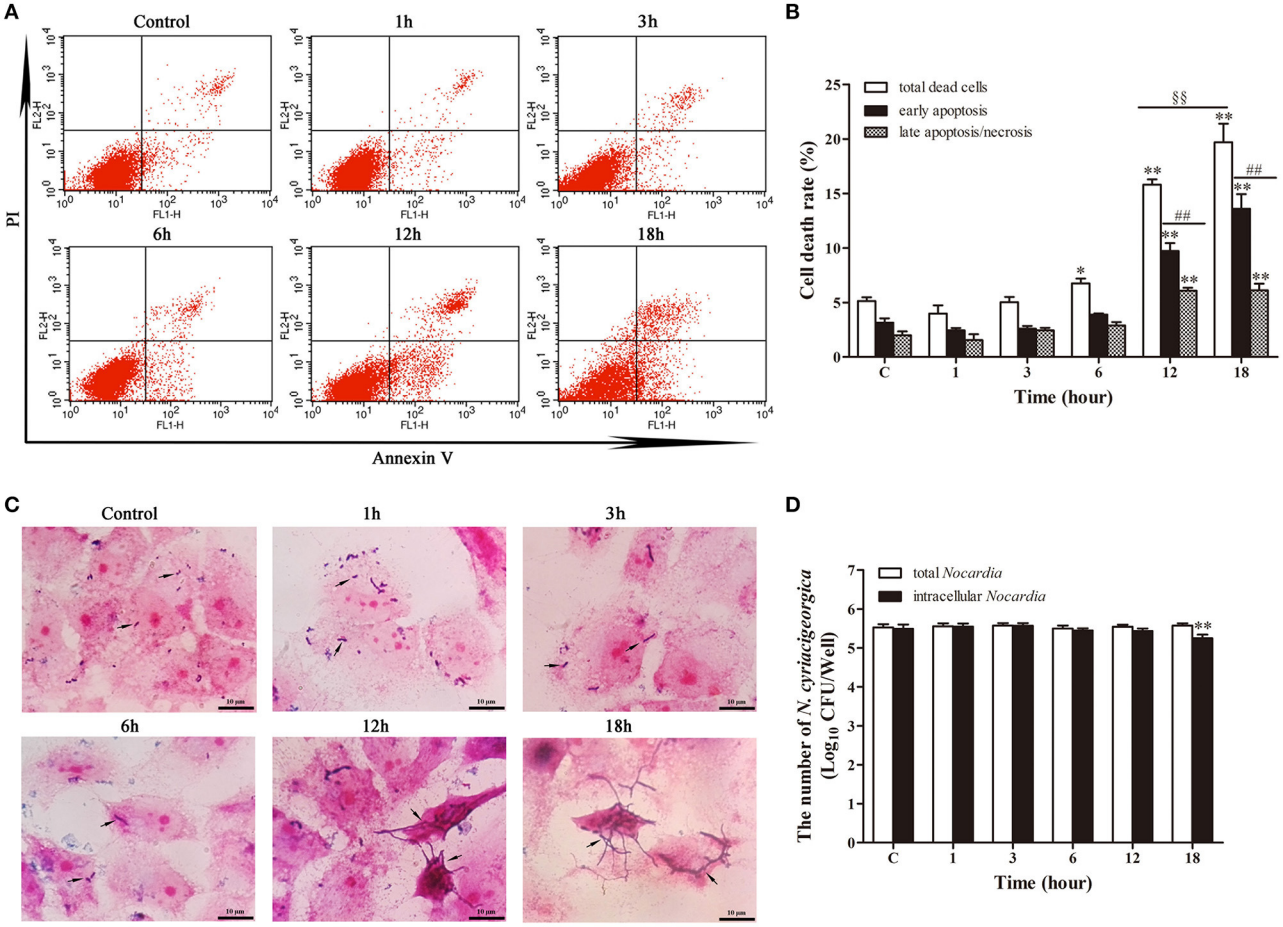
**Apoptosis analysis in parallel with the growth of intracellular *N. cyriacigeorgica*. (A)** Two dimensional scatter plots of FITC Annexin V vs. PI through flow cytometry. **(B)** Percentage of early apoptotic cells and late apoptotic/necrotic cells. **(C)** Gram staining of bMECs and *Nocardia* under light microscope. From 1–6 h, only few short rods of *Nocardia* can be observed (black arrows); while from 12 to 18 h, short rods grew into long mycelia (black arrows). **(D)** The number of total *Nocardia* and intracellular *Nocardia*. Data were presented as Mean ± *SD* of at least three independent experiments. ^*^P < 0.05, ^**^P < 0.01 as compared with the control group. ^*##*^*P* < 0.01 as compared between the rate of early and late apoptosis/necrosis at the same time point. ^§§^*P* < 0.01 represents the significant differences in total cells death rate between 12 and 18 h.

The growth of intracellular *Nocardia* showed that the apoptosis rate was increased significantly (*p* < 0.01) from 12 h compared with control group (Figures 8A,B). At 12 and 18 h, most of the dead cells were at the early apoptotic stage (*p* < 0.01). In addition, the total death rate of bMECs at 18 h (19.70%) was significantly higher (*p* < 0.01) than at 12 h (15.80%). Strikingly, Gram staining showed that the *Nocardia* were short rod-shaped with no considerable growth during 1–6 h; nevertheless, they germinated into unique long mycelial form at 12 and 18 h, leading to patho-morphological changes of infected cells (Figure 8C). Bacterial counting presented an interesting results that there were no significant difference of the number of total *Nocardia* at different incubation time compared with control group. Similar results for the enumeration of intracellular *Nocardia* were also found within 12 h. This was in accordance with the apoptosis analysis, which presented that the apoptosis significantly increased at 12 and 18 h. The intracellular bacteria significantly decreased (*p* < 0.01) at 18 h compared with control group (Figure 8D). The unchanging number of *Nocardia* and the results of Gram staining revealed that both intracellular and extracellular *N. cyriacigeorgica* grew from small rods to mycelia rather than to reproduce during 18 h incubation (Figures 8C,D). During the robust growth phase of *Nocardia* at 12–18 h, cell apoptosis was aggravated remarkably (Figures 8A–C).

## Discussion

Bacterial adhesion and invasion played important roles in establishing infection. The quantitative analysis of adhesion and invasion of *N. cyriacigeorgica* and visualized results of SEM and TEM showed that *N. cyriacigeorgica* possess the adhesion and penetration abilities. Significantly, we observed that *N. cyriacigeorgica* could promptly adhere to and penetrated into the bMECs within 10 min. These results were in accordance with a previous study which reported 68% adherence rate and 70.8% penetration rate of *N. asteroides* GUH-2 (*N. cyriacigeorgica* GUH-2) to endothelial cells in brain within 15–25 min exposure period (Beaman and Ogata, 1993). The findings of this study and some previous studies suggested that *Nocardia* has the capability of adhesion, invasion and viability within various host cells both *in vitro* and *in vivo* (Beaman and Ogata, 1993; Beaman and Beaman, 1994, 1998).

In the log-phase *N. cyriacigeorgica* depicted strong penetrative and adhesion ability than the stationary-phase and only live log-phase organisms can penetrate into cells (Beaman and Beaman, 1998). *Nocardia* can adhere to cells through surface adhesion molecules, live, and heat-killed *Nocardia* both can bind to the cell surface. Most of studies demonstrated that a filamentous tip associated 43-kDa protein of *N. asteroides* GUH-2 played a dominant role in attachment and invasion to pulmonary epithelial cells and HeLa cells (Beaman and Beaman, 1998). The antiserum against the 43-kDa antigen inhibited apical adhesion and penetration to pulmonary epithelial cells, and prevented spread to the brain. Through gene sequence analysis of *N. cyriacigeorgica*, a series of putative mammalian cell entry proteins (mce) of *N. cyriacigeorgica* (Vera-Cabrera et al., 2013; Zoropogui et al., 2013) were found and these proteins were essential for pathogens to attach, enter and survive in the host cells (Zhang and Xie, 2011). Additionally, adhesion of *Nocardia* was associated with the cell surface structures, such as microvilli. Cell surface provides a direct connection with the bacteria. As evident from the SEM results, this presented that the microvilli of bMECs can capture *Nocardia*. This phenomenon was also observed previously on other cells (Beaman and Beaman, 1994, 1998).

After exposure to bMECs, *N. cyriacigeorgica* infection led to perforation in cell membranes, release of LDH, fragmentation of nuclear membrane, chromatin condensation and mitochondrial degeneration, which can caused untimely collapse of the entire cell. Previous studies suggested that the pathogenicity of *Nocardia* is dependent on the virulence factors and toxins. The putative virulence factors of *N. cyriacigeorgica* included catalase, superoxide dismutase, hemolysin, invasion, protease, mammalian cell proteins (mec), mycolic acids, nitrate reductase, and PE/PPE/PGRS family proteins through gene analysis (Vera-Cabrera et al., 2013; Zoropogui et al., 2013), among most of them had been proved. Catalase and superoxide dismutase of *N. cyriacigeorgica* probably acted important roles in defense against deleterious superoxide and reactive oxygen species (ROS) during intracellular killing by phagocytes (Wu et al., 2006). The presence of nitrate reductase for *N. cyriacigeorgica* suggested an ability to grow under low-oxygen conditions in stimulated macrophages (Zoropogui et al., 2013). Significantly, mycolic acids, the major and specific lipid components of cell envelope, have important implications in the pathogenesis of *Mycobacterium, Nocardia* and *Rhodococcus*, and *Corynebacterium* (Elamin et al., 2012; Verschoor et al., 2012). The cell wall-associated lipids of *Nocardia* induced the production of the proinflammatory cytokines and inhibited important macrophage microbicidal effects (Trevino-Villarreal et al., 2012).

Bacteria often elicited cell apoptosis as a survival strategy. Pathogens have evolved a series of toxins and virulence factors to modulate host cell death (Lamkanfi and Dixit, 2010). During the first 6 h of *Nocardia* infection, there were only several short nocardial filaments on the cell surface imparting mild morphological changes in bMECs, and there were also no obvious cytotoxic effects and ΔΨm, following no or low number of dead cells. Nevertheless, during the late stage of infection, short nocardial mycelia grew into long and strong mycelia, which can invade and penetrate into the bMECs, and even grew within cells; simultaneously, *N. cyriacigeorgica* was found to significantly elicit cell apoptosis, and exacerbated changes in cell morphology and ultrastructure, with a typical fragmentation of DNA and a collapse of ΔΨm from 12 h. Besides, there was robust release of LDH from 12 h, which was associated with the serious cell membrane breakage and the large number of late apoptotic/necrotic cell in this period. A previous study demonstrated that live *Nocardia* can invade into cells and significantly increase in apoptosis rate in infected cells compared to heat-killed *Nocardia* (Barry and Beaman, 2007). Considering these above results, we can assume that the penetration and aggressive growth of *N. cyriacigeorgica* mycelia might be one of the factors to induce death of bMECs. Additionally, on PC12 cells, *Nocardia* culture filtrate was found to induce apoptotic morphology after 24 h-treatment and increased release of LDH after 48 h (Loeffler et al., 2004), indicated that the metabolites of *Nocardia* are toxic to cells.

An extra experiment was performed to know the cytopathic and apoptotic effects of only intracellular *Nocardia*, which was coupled with study regarding the growth of intracellular bacteria. For this purpose the extracellular bacteria were killed with amikacin and apopotosis and growth of the intracellular bacteria was evaluated. Interesting, the results showed that apoptosis and bacterial growth have a parallel relationship. These effects were significantly exhibited during 12 and 18 h. Importantly, there was no significant changes in the total number of *Nocardia* but the bacteria grew into mycelial form, imparting cell damage and apoptosis. At 12 and 18 h of infection, a rapid growth of *Nocardia* was noted without reproduction, which indicated that the aggressive growth of intracellular *Nocardia* may act as an important role in the cell injury leading to cell death.

These data suggested that *N. cyriacigeorgica* infection and invasion can induce both apoptotic and necrotic changes in bMECs after 6 h, corroborating with the previous findings in which *Nocardia* induced apoptosis in PC12 cells and HeLa cells (Tam et al., 2002; Loeffler et al., 2004; Barry and Beaman, 2007). In Hela and PC12 cells, live *Nocardia* promoted a significant DNA fragmentation during 6 h-exposure period (Tam et al., 2002; Barry and Beaman, 2007). Previous studies also showed that *Nocardia* localized in substantia nigra and then underwent a rapid growth, inducing apoptosis of dopaminergic cells and failure of inflammatory response in 24 h (Kohbata and Beaman, 1991; Tam et al., 2002). Cell apoptosis is a double-edged sword both for host and bacteria during infection. For the host, cell apoptosis is a defense mechanism against pathogenic bacteria to prevent the release of intracellular bacteria and the spread of bacteria. Whereas, for bacteria, they induce cell apoptosis to block the spilling of cellular contents, to suppress the motivation of inflammatory reaction and then to evade host defenses. Several bacterial pathogens can cause cell apoptosis; whereas, many intracellular pathogens, such as *Mycobacterium tuberculosis*, can protect infected cells from apoptosis for its survival (Faherty and Maurelli, 2008; Butler et al., 2012). However, in the process of infection, *N. cyriacigeorgica*, being an intracellular pathogen, caused apoptosis and necrosis in bMECs.

**Figure 9:**
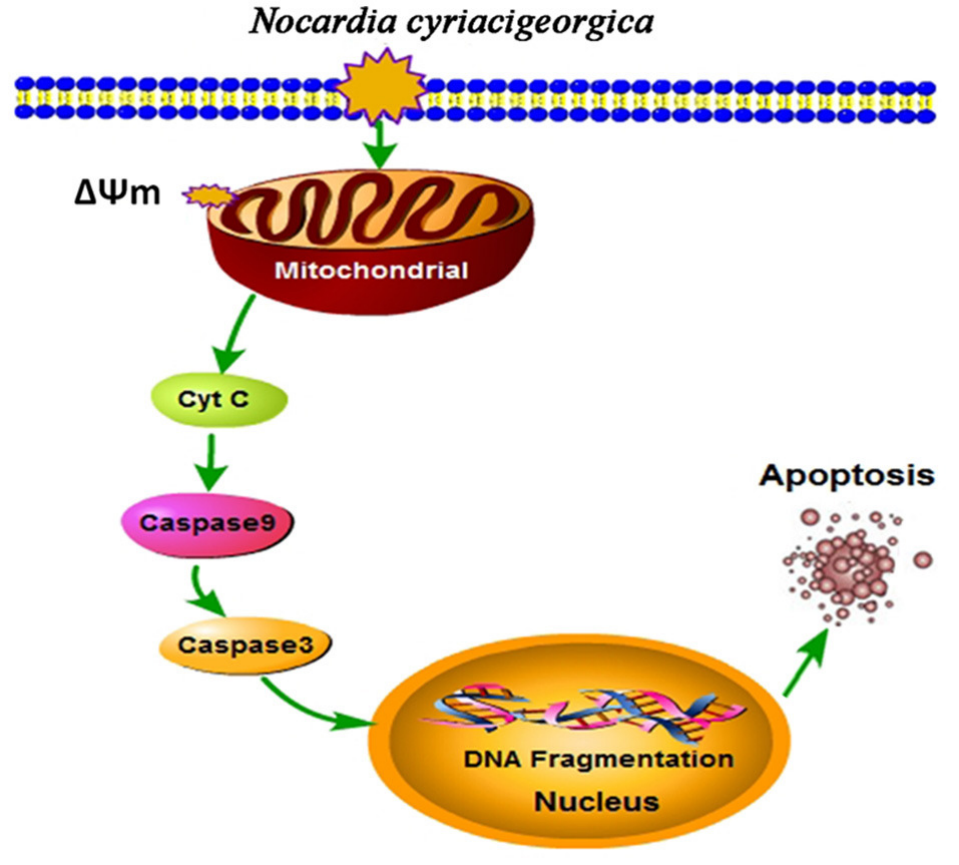
**Schematic illustration of mitochondrial-caspase induced apoptosis**. Depolarization of mitochondrial transmembrane (ΔΨm) causes release of cytochrome c (Cyt C), which may initiate caspase cascade. Cyt C bonds with apoptotic protease-activating factor 1 (Apaf-1) (not shown in the Figure) and activates caspase-9, this cleaves and activates caspase-3, which triggers the apoptosis. Translocation of endonuclease G (Endo G) from the mitochondria to the nucleus and nuclear activation of DNA fragmentation factor (DFF) (not shown in the Figure) caused by caspase activation induced nucleosomal DNA fragmentation during apoptosis (Kitazumi and Tsukahara, 2011).

The ultrastructural pathology of TEM showed the mitochondrial damage. To further validate our results, the collapse of ΔΨm and release of mitochondrial cytochrome c were determined, presenting a decrease of ΔΨm and an increase of cytochrome c release. On the other hand, western blot analysis suggested the promotion of casepase-9 and casepase-3 activation. Likewise, similar effects on mitochondria and caspase-3 activity have been proved in the *Nocardia*-induced Hela apoptosis at a MOI of 5:1co-cultured for 5 h (Barry and Beaman, 2007). Altogether, from these findings we can draw the conclusion that *N. cyriacigeorgica* can induce apoptosis of bMECs mediated by a mitochondria-caspase dependent pathway. Mitochondrial dysfunction to release proteins from the intermembrane space into the cytosol is the pivotal focus in the process of apoptosis (Wang and Youle, 2009). As for apoptotic signaling, cytochrome c was a critical apoptogenic factor and was capable of initiating the caspase cascade (Kagan et al., 2009). Normally, cytochrome c is localized in the mitochondrial intermembrane space. During the infection of *N. cyriacigeorgica*, the opening of the mitochondrial permeability transition pore induced depolarization of mitochondrial transmembrane ΔΨm, release of apoptogenic factors and loss of oxidative phosphorylation (Tait and Green, 2010). Cytochrome c transposed from the mitochondrial membrane space to the cytosol; then bond to apoptotic protease-activating factor 1 (Apaf-1), triggered the formation of the apoptosome, inducing recruitment and activation of caspase-9. Caspase-9 cleaves and activates executioner caspase-3, which triggers the apoptosis (Ow et al., 2008; Tait and Green, 2010). Simultaneously, translocation of endonuclease G (Endo G) from the mitochondria to the nucleus and nuclear activation of DNA fragmentation factor (DFF) caused by caspase activation induced nucleosomal DNA fragmentation during apoptosis (Kitazumi and Tsukahara, 2011). DNA fragmentation is a hallmark of apoptosis and is one of the last consequences of apoptosis (Kitazumi and Tsukahara, 2011). The major schematic representation of mitochondrial-caspase induced apoptotic pathway studied in this work is elucidated in Figure 9.

In summary, these results of the present study demonstrated that clinical *N. cyriacigeorgica* was able to adhere and invade the bMECs, causing disruption of cell membrane and mitochondrial degeneration. Our data supports the hypothesis that *N. cyriacigeorgica* induced host cell death via an apoptotic mechanism and the later stage of *N. cyriacigeorgica* infection followed mitochondrial-dependent apoptotic pathway in bMECs. These findings provide that the apoptosis of bMECs was induced via triggering the caspase cascade and might present important insights into the mechanisms of *N. cyriacigeorgica* infections in bovine mammary tissues.

## Ethics statement

The present study was conducted in accordance with the ethical guide lines of China Agricultural University (CAU), Beijing. Furthermore, prior to the initiation of this work, proper approval was granted by the departmental committee of College of Veterinary Medicine, CAU.

## Author contributions

WC planned, performed the experiments, and then wrote manuscript. XG and YL performed the experiments, LZ and TA helped in analysis of flow cytometry experiments, GL collaborated in writing manuscript, MS and TA revised and corrected the manuscript, JG analyzed the data and BH designed and evaluated the research. All authors have read and approved the final manuscript.

## Funding

This research was supported by Ministry of Education in China major project (No. 313054) and the National Natural Science Foundation of China (No. 3151101034 and NO. 31572587).

### Conflict of interest statement

The authors declare that the research was conducted in the absence of any commercial or financial relationships that could be construed as a potential conflict of interest.

## References

[B1] Al Akhrass F. Hachem R. Mohamed J. A. Tarrand J. Kontoyiannis D. P. Jyotsna C.. (2011). Central venous catheter-associated *Nocardia* bacteremia in cancer patients. Emerg. Infect. Dis. 17, 1651–1658. 10.3201/eid1709.101810 21888790 PMC3322064

[B2] Ambrosioni J. Lew D. Garbino J. (2010). Nocardiosis: updated clinical review and experience at a tertiary center. Infection 38, 89–97. 10.1007/s15010-009-9193-9 20306281

[B3] Ashida H. Mimuro H. Ogawa M. Kobayashi T. Sanada T. Kim M. (2011). Cell death and infection: a double-edged sword for host and pathogen survival. J. Cell Biol. 195, 931–942. 10.1083/jcb.201108081 22123830 PMC3241725

[B4] Barry D. P. Beaman B. L. (2007). *Nocardia asteroides* strain GUH-2 induces proteasome inhibition and apoptotic death of cultured cells. Res. Microbiol. 158, 86–96. 10.1016/j.resmic.2006.11.001 17258894 PMC1831872

[B5] Bättig U. Wegmann P. Meyer B. Penseyres J. (1989). *Nocardia* mastitis in cattle. 1. Clinical observations and diagnosis in 7 particular cases. Schweizer Archiv. Tierheilkunde 132, 315–322. 2205001

[B6] Bawa B. Bai J. Whitehair M. Purvis T. DeBey B. M. (2010). Bovine abortion associated with *Nocardia farcinica*. J. Vet. Diagn. Invest. 22, 108–111. 10.1177/104063871002200122 20093696

[B7] Bayles K. W. Wesson C. A. Liou L. E. Fox L. K. Bohach G. A. Trumble W. R. (1998). Intracellular *staphylococcus aureus* escapes the endosome and induces apoptosis in epithelial cells. Infect. Immun. 66, 336–342. 9423876 10.1128/iai.66.1.336-342.1998 PMC107895

[B8] Beaman B. L. Beaman L. (1994). *Nocardia* species: host-parasite relationships. Clin. Microbiol. Rev. 7, 213–264. 10.1128/CMR.7.2.213 8055469 PMC358319

[B9] Beaman B. L. Beaman L. (1998). Filament tip-associated antigens involved in adherence to and invasion of murine pulmonary epithelial cells *in vivo* and HeLa cells *in vitro* by *Nocardia asteroides*. Infect. Immun. 66, 4676–4689. 9746564 10.1128/iai.66.10.4676-4689.1998 PMC108575

[B10] Beaman B. L. Ogata S. A. (1993). Ultrastructural analysis of attachment to and penetration of capillaries in the murine pons, midbrain, thalamus, and hypothalamus by *Nocardia asteroides*. Infect. Immun. 61, 955–965. 8381774 10.1128/iai.61.3.955-965.1993 PMC302825

[B11] Beaman B. L. Sugar A. M. (1983). *Nocardia* in naturally acquired and experimental infections in animals. J. Hyg. 91, 393–419. 10.1017/S0022172400060447 6363525 PMC2129343

[B12] Beaman B. L. Tam S. (2008). An unusual murine behavior following infection with log-phase *Nocardia asteroides* type 6 strain GUH-2 (*Nocardia cyriacigeorgica* GUH-2). Microbes Infect. 10, 840–843. 10.1016/j.micinf.2008.04.007 18538618

[B13] Brown J. M. Cowley K. D. Manninen K. I. McNeil M. M. (2007). Phenotypic and molecular epidemiologic evaluation of a *Nocardia farcinica* mastitis epizootic. Vet. Microbiol. 125, 66–72. 10.1016/j.vetmic.2007.04.044 17553640

[B14] Brown-Elliott B. A. Conville P. Wallace R. J. (2015). Current status of *Nocardia* taxonomy and recommended identification methods. Clin. Microbiol. Newslett. 37, 25–32. 10.1016/j.clinmicnews.2015.01.007

[B15] Butler R. E. Brodin P. Jang J. Jang M. S. Robertson B. D. Gicquel B. (2012). The balance of apoptotic and necrotic cell death in *Mycobacterium tuberculosis* infected macrophages is not dependent on bacterial virulence. PLoS ONE 7:e47573 10.1371/journal.pone.0047573 23118880 PMC3484146

[B16] Chapman G. Beaman B. L. Loeffler D. A. Camp D. M. Domino E. F. Dickson D. W.. (2003). *In situ* hybridization for detection of nocardial 16S rRNA: reactivity within intracellular inclusions in experimentally infected cynomolgus monkeys—and in Lewy body-containing human brain specimens. Exp. Neurol. 184, 715–725. 10.1016/s0014-4886(03)00337-6 14769363

[B17] Condas L. A. Ribeiro M. G. Yazawa K. de Vargas A. P. C. Salerno T. Giuffrida R.. (2013). Molecular identification and antimicrobial susceptibility of *Nocardia* spp. isolated from bovine mastitis in Brazil. Vet. Microbiol. 167, 708–712. 10.1016/j.vetmic.2013.08.019 24060098

[B18] Conville P. S. Witebsky F. G. (2011). Nocardia, Rhodococcus, Gordonia, Actinomadura, Streptomyces, and other aerobic actinomycetes, in Manual of Clinical Microbiology, 10th Ed (Washington, DC: ASM Press), 443–471.

[B19] Cook J. Holliman A. (2004). Mastitis due to *Nocardia asteroides* in a UK dairy herd following restocking after FMD. Vet. Rec. 154, 267–268. 10.1136/vr.154.9.267 15029966

[B20] Dego O. K. Van Dijk J. E. Nederbragt H. (2002). Factors involved in the early pathogenesis of bovine *Staphylococcus aureus* mastitis with emphasis on bacterial adhesion and invasion. A review. Vet. Q. 24, 181–198. 10.1080/01652176.2002.9695135 12540135

[B21] Dohoo I. (1989). *Nocardia* spp. mastitis in Canada. Can. Vet. J. 30, 969. 17423480 PMC1681337

[B22] Elamin A. A. Stehr M. Singh M. (2012). Lipid droplets and *Mycobacterium leprae* infection. J. Pathog. 2012:361374. 10.1155/2012/361374 23209912 PMC3503283

[B23] Elmore S. (2007). Apoptosis: a review of programmed cell death. Toxicol. Pathol. 35, 495–516. 10.1080/01926230701320337 17562483 PMC2117903

[B24] Faherty C. S. Maurelli A. T. (2008). Staying alive: bacterial inhibition of apoptosis during infection. Trends Microbiol. 16, 173–180. 10.1016/j.tim.2008.02.001 18353648 PMC2746948

[B25] Galluzzi L. Vitale I. Abrams J. M. Alnemri E. S. Baehrecke E. H. Blagosklonny M. V.. (2012). Molecular definitions of cell death subroutines: recommendations of the Nomenclature Committee on Cell Death 2012. Cell Death Differ. 19, 107–120. 10.1038/cdd.2011.96 21760595 PMC3252826

[B26] Hamid M. E. (2012). Epidemiology, pathology, immunology and diagnosis of bovine farcy: a review. Prev. Vet. Med. 105, 1–9. 10.1016/j.prevetmed.2012.01.004 22341733

[B27] Hamid M. El Sanousi S. Minnikin D. Goodfellow M. (1998). Isolation of *Nocardia farcinica* from zebu cattle suffering from mastitis in Sudan. Sudan J. Vet. Sci. Anim. Husb. 37, 66–71.

[B28] Hashemi-Shahraki A. Heidarieh P. Bostanabad S. Z. Hashemzadeh M. Feizabadi M. M. Schraufnagel D.. (2015). Genetic diversity and antimicrobial susceptibility of *Nocardia* species among patients with nocardiosis. Sci. Rep. 5:17862. 10.1038/srep17862 26638771 PMC4671095

[B29] Holland S. M. (2010). Chronic granulomatous disease. Clin. Rev. Allergy Immunol. 38, 3–10. 10.1007/s12016-009-8136-z 19504359

[B30] Kagan V. E. Bayır H. A. Belikova N. A. Kapralov O. Tyurina Y. Y. Tyurin V. A.. (2009). Cytochrome c/cardiolipin relations in mitochondria: a kiss of death. Free Radic. Biol. Med. 46, 1439–1453. 10.1016/j.freeradbiomed.2009.03.004 19285551 PMC2732771

[B31] Kitazumi I. Tsukahara M. (2011). Regulation of DNA fragmentation: the role of caspases and phosphorylation. FEBS J. 278, 427–441. 10.1111/j.1742-4658.2010.07975.x 21182594

[B32] Kohbata S. Beaman B. L. (1991). L-dopa-responsive movement disorder caused by *Nocardia asteroides* localized in the brains of mice. Infect. Immun. 59, 181–191. 1670928 10.1128/iai.59.1.181-191.1991 PMC257724

[B33] Kohbata S. Emura S. Kadoya C. (2009). Filterable forms of *Nocardia*: a preferential site of infection in the mouse brain. Microbes Infect. 11, 744–752. 10.1016/j.micinf.2009.04.013 19376258

[B34] Lamkanfi M. Dixit V. M. (2010). Manipulation of host cell death pathways during microbial infections. Cell Host Microbe 8, 44–54. 10.1016/j.chom.2010.06.007 20638641

[B35] Lira R. M. M. Flores A. Y. L. Carmona M. C. S. Stern A. O. (2016). Experimental granulomatous pulmonary nocardiosis in BALB/C Mice. PLoS ONE 11:e0157475 10.1371/journal.pone.0157475 27303806 PMC4909231

[B36] Liu W. L. Lai C. C. Ko W. C. Chen Y. H. Tang H. J. Huang Y. L.. (2011). Clinical and microbiological characteristics of infections caused by various *Nocardia* species in Taiwan: a multicenter study from 1998 to 2010. Eur. J. Clin. Microbiol. Infect. Dis. 30, 1341–1347. 10.1007/s10096-011-1227-9 21461846

[B37] Loeffler D. A. Camp D. M. Qu S. Beaman B. L. LeWitt P. A. (2004). Characterization of dopamine-depleting activity of *Nocardia asteroides* strain GUH-2 culture filtrate on PC12 cells. Microb. Pathog. 37, 73–85. 10.1016/j.micpath.2004.05.001 15312847

[B38] Meester I. Rosas-Taraco A. G. Salinas-Carmona M. C. (2014). *Nocardia brasiliensis* induces formation of foamy macrophages and dendritic cells *in vitro* and *in vivo*. PLoS ONE 9:e100064. 10.1371/journal.pone.0100064 24936860 PMC4061056

[B39] Ow Y. P. Green D. R. Hao Z. Mak T. W. (2008). Cytochrome c: functions beyond respiration. Nat. Rev. Mol. Cell. Biol. 9, 532–542. 10.1038/nrm2434 18568041

[B40] Pereyra E. A. Picech F. Renna M. S. Baravalle C. Andreotti C. S. Russi R.. (2016). Detection of *Staphylococcus aureus* adhesion and biofilm-producing genes and their expression during internalization in bovine mammary epithelial cells. Vet. Microbiol. 183, 69–77. 10.1016/j.vetmic.2015.12.002 26790937

[B41] Pisoni G. Locatelli C. Alborali L. Rosignoli C. Allodi S. Riccaboni P.. (2008). Short communication: outbreak of *Nocardia neocaledoniensis* mastitis in an Italian dairy herd. J. Dairy Sci. 91, 136–139. 10.3168/jds.2007-0477 18096934

[B42] Pöhlmann-Dietze P. Ulrich M. Kiser K. B. Döring G. Lee J. C. Fournier J. M.. (2000). Adherence of *Staphylococcus aureus* to endothelial cells: influence of capsular polysaccharide, global regulatoragr, and bacterial growth phase. Infect. Immun. 68, 4865–4871. 10.1128/IAI.68.9.4865-4871.2000 10948098 PMC101683

[B43] Ribeiro M. G. Salerno T. Mattos-Guaraldi A. L. D. Camello T. C. F. Langoni H. Siqueira A. K.. (2008). Nocardiosis: an overview and additional report of 28 cases in cattle and dogs. Rev. Inst. Med. Trop. Sao Paulo 50, 177–185. 10.1590/S0036-46652008005000004 18516465

[B44] Sullivan D. C. Chapman S. W. (2010). Bacteria that masquerade as fungi: actinomycosis/nocardia. Proc. Am. Thorac. Soc. 7, 216–221. 10.1513/pats.200907-077AL 20463251

[B45] Tait S. W. Green D. R. (2010). Mitochondria and cell death: outer membrane permeabilization and beyond. Nat. Rev. Mol. Cell Biol. 11, 621–632. 10.1038/nrm2952 20683470

[B46] Tam S. Barry D. P. Beaman L. Beaman B. L. (2002). Neuroinvasive *Nocardia asteroides GUH-2* induces apoptosis in the substantia nigra *in vivo* and dopaminergic cells *in vitro*. Exp. Neurol. 177, 453–460. 10.1006/exnr.2002.8012 12429191

[B47] Trevino-Villarreal J. H. Vera-Cabrera L. Valero-Guillén P. L. Salinas-Carmona M. C. (2012). *Nocardia brasiliensis* cell wall lipids modulate macrophage and dendritic responses that favor development of experimental actinomycetoma in BALB/c mice. Infect. Immun. 80, 3587–3601. 10.1128/IAI.00446-12 22851755 PMC3457583

[B48] Vera-Cabrera L. Ortiz-Lopez R. Elizondo-Gonzalez R. Ocampo-Candiani J. (2013). Complete genome sequence analysis of *Nocardia brasiliensis* HUJEG-1 reveals a saprobic lifestyle and the genes needed for human pathogenesis. PLoS ONE 8:e65425. 10.1371/journal.pone.0065425 23755230 PMC3670865

[B49] Verschoor J. A. Baird M. S. Grooten J. (2012). Towards understanding the functional diversity of cell wall mycolic acids of *Mycobacterium tuberculosis*. Prog. Lipid Res. 51, 325–339. 10.1016/j.plipres.2012.05.002 22659327

[B50] Viguier C. Arora S. Gilmartin N. Welbeck K. O'kennedy R. (2009). Mastitis detection. Current trends and future perspectives. Trends Biotechnol. 27, 486–493. 10.1016/j.tibtech.2009.05.004 19616330

[B51] Wang C. Youle R. J. (2009). The role of mitochondria in apoptosis. Annu. Rev. Genet. 43, 95. 10.1146/annurev-genet-102108-134850 19659442 PMC4762029

[B52] Wilson J. W. (2012). Nocardiosis: updates and clinical overview. Mayo Clin. Proc. 87, 403–407. 10.1016/j.mayocp.2011.11.016 22469352 PMC3498414

[B53] Wu G. Nie L. Zhang W. (2006). Predicted highly expressed genes in *Nocardia farcinica* and the implication for its primary metabolism and nocardial virulence. Antonie Van Leeuwenhoek 89, 135–146. 10.1007/s10482-005-9016-z 16496092

[B54] Xu Z. Y. Zheng M. X. Zhang Y. Cui X. Z. Yang S. S. Liu R. L.. (2016). The effect of the mitochondrial permeability transition pore on apoptosis in *Eimeria tenella* host cells. Poult. Sci. 95, 2405–2413. 10.3382/ps/pew198 27444446

[B55] Yang S. S. Zheng M. X. Xu H. C. Cui X. Z. Zhang Y. Zhao W. L.. (2015). The effect of mitochondrial ATP-sensitive potassium channels on apoptosis of chick embryo cecal cells by *Eimeria tenella*. Res. Vet. Sci. 99, 188–195. 10.1016/j.rvsc.2015.02.002 25744434

[B56] Zhang F. Xie J. P. (2011). Mammalian cell entry gene family of *Mycobacterium tuberculosis*. Mol. Cell. Biochem. 352, 1–10. 10.1007/s11010-011-0733-5 21258845

[B57] Zoropogui A. Pujic P. Normand P. Barbe V. Belli P. Graindorge A.. (2013). The *Nocardia cyriacigeorgica* GUH-2 genome shows ongoing adaptation of an environmental Actinobacteria to a pathogen's lifestyle. BMC Genomics 14:286. 10.1186/1471-2164-14-286 23622346 PMC3751702

